# Granulomatous dermatitis: a rare pitfall in lymphoma staging with [^18^F]FDG-PET/CT

**DOI:** 10.1007/s00259-023-06284-3

**Published:** 2023-05-26

**Authors:** Johanna S. Enke, Alexander Gäble, Nic G. Reitsam, Tina Schaller, Martin Trepel, Klaus Hirschbühl, Julia Welzel, Alexander Dierks, Malte Kircher, Constantin Lapa

**Affiliations:** 1 Nuclear Medicine, Faculty of Medicine, University of Augsburg, Augsburg, Germany; 2 Pathology, Faculty of Medicine, University of Augsburg, Augsburg, Germany; 3 Hematology and Oncology, Faculty of Medicine, University of Augsburg, Augsburg, Germany; 4 Dermatology, Faculty of Medicine, University of Augsburg, Augsburg, Germany

A 36-year-old male with anaplastic large T cell lymphoma (A; nodal manifestations in the left iliac and left inguinal region, arrow) who had received six cycles of chemotherapy (brentuximab vedotin, cyclophosphamide, doxorubicin, and prednisone) presented for restaging prior to autologous stem cell transplantation. A few days earlier, the patient had noticed multiple new tender subcutaneous nodules, and erythemato-squamous, polymorphous, partially atrophic plaques all over his body (B1). [^18^F]FDG-positron emission tomography/computed tomography (PET/CT) showed complete metabolic response of primary lymphoma manifestations but revealed intense tracer accumulation in the disseminated subcutaneous nodules (B, axial image B2). Besides cutaneous involvement by T cell lymphoma, differential diagnoses included cutaneous sarcoidosis and pityriasis rosea. Biopsy of a subcutaneous nodule revealed no evidence of malignancy but granulomatous inflammation (B3) most consistent with reactive granulomatous dermatitis. After initiation of prednisone therapy, all (sub-)cutaneous lesions quickly resolved, and the patient was eligible for stem cell transplantation. Follow-up [^18^F]FDG-PET/CT demonstrated only residual tracer uptake of some lesions and a sustained complete lymphoma response (C).

Reactive granulomatous dermatitis is a very rare skin disease with only several hundred cases reported worldwide so far [1], most commonly associated with autoimmune disorders such as rheumatoid arthritis. Furthermore, it has been associated with hematologic malignancies, including — in approximately 3% of cases — (B cell) lymphoma [1–3]. To our knowledge, this is one of the very first cases of granulomatous dermatitis in anaplastic large T cell lymphoma [4], and the first visualization of granulomatous dermatitis by [^18^F]FDG-PET/CT mimicking cutaneous lymphoma manifestations.
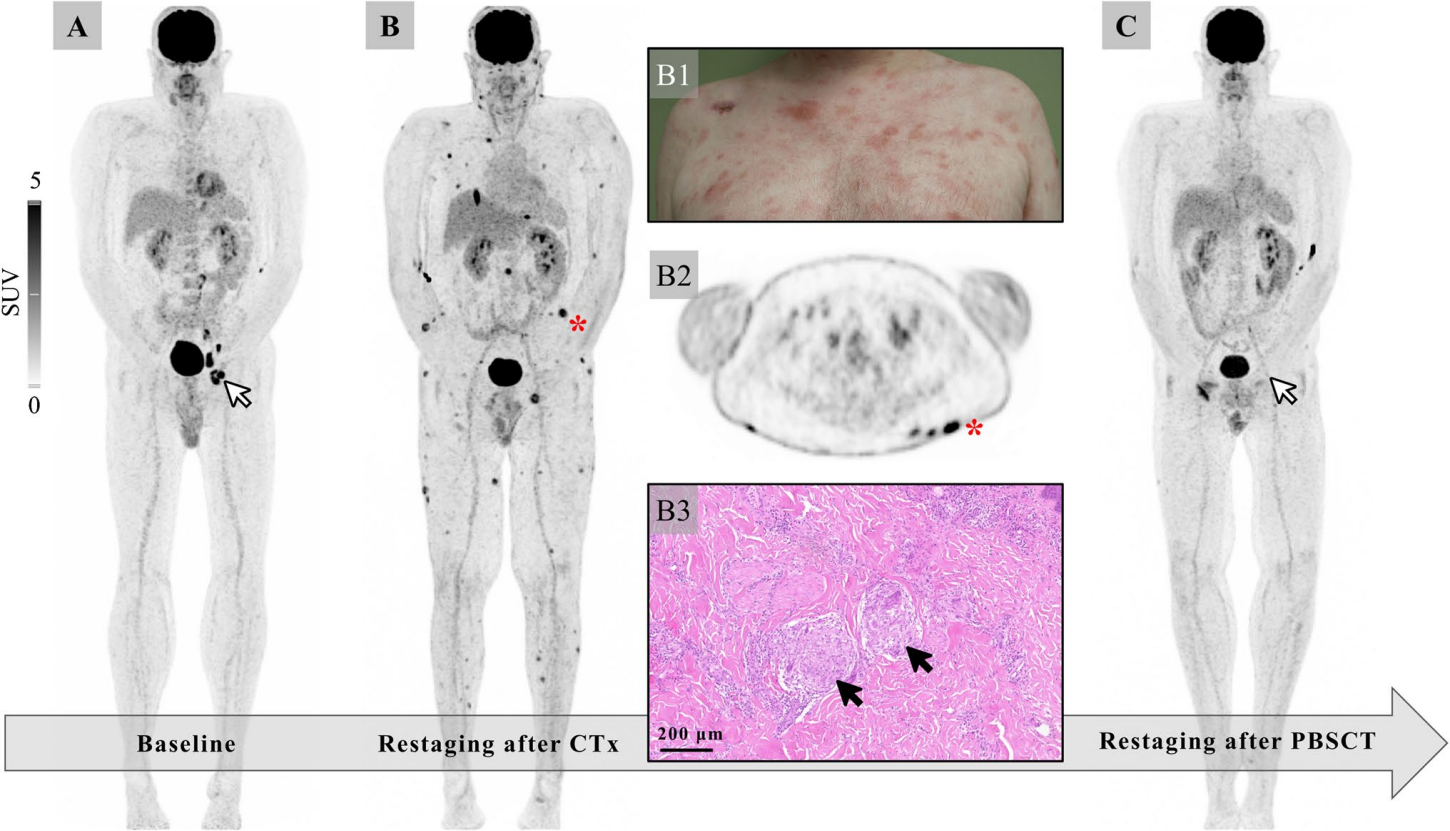


## Data Availability

The data that support the findings of this study are available from the corresponding author, [JSE], upon reasonable request.
